# Accommodative changes after SMILE for moderate to high myopia correction

**DOI:** 10.1186/s12886-016-0352-8

**Published:** 2016-10-04

**Authors:** Ke Zheng, Tian Han, Xingtao Zhou

**Affiliations:** Key Lab of Myopia, Ministry of Health, Department of Ophthalmology, Eye & ENT Hospital of Fudan University, 19 Baoqing Road, Shanghai, 200031 China

**Keywords:** Small incision lenticule extraction (SMILE), Accommodative response, Accommodative lag, Visual discomfort symptom

## Abstract

**Background:**

To investigate accommodative response and accommodative lag changes after femtosecond laser small incision lenticule extraction (SMILE) for moderate to high myopia correction.

**Methods:**

A total of 32 eyes of 32 patients with no strabismus who underwent SMILE were enrolled in this prospective clinical study. The accommodative response was obtained viewing monocularly with spherical equivalent refractive error corrected, using an open-field autorefractor at different stimulus levels (2.00D, 2.50D, 3.00D, 4.00D and 5.00D) for the right eye before a standard SMILE surgery and at 1-month follow-up after surgery.

**Results:**

The mean age of the patients were 23.34 ± 2.90 years and the mean preoperative manifest refraction spherical equivalent was −5.74 ± 1.98 diopters. Significant differences were detected in both preoperative and postoperative accommodative responses to different stimulus levels (*P* < 0.001). Multiple linear regression model analysis revealed preoperative manifest refractive spherical equivalent (*P* = 0.006) and preoperative accommodative lag (*P* = 0.04) showed a significant impact on postoperative accommodative lag.

**Conclusions:**

This is the first report of accommodative changes after SMILE. Our preliminary results showed that a decrease in postoperative accommodative lag that might be related to the relief of the visual discomfort symptom.

## Background

Femtosecond laser small incision lenticule extraction (SMILE) was first introduced by Shah and Sekundo in 2011 and improved with continuous curvilinear lenticulerrhexis (CCL) technique by Zhou in 2015 [1–3]. SMILE is an effective and safe refractive surgery with a small incision and flapless feature [4]. Accommodation is an old theme in visual refraction. Accommodation is the ability to adjust the refractive power of the eye to bring the conjugate focus of the retina identical to an object. The accommodative lag refers to the insufficient accommodation and provides information about the accuracy of an individual’s accommodative system handles increased demand. The accommodative lag has been investigated to have a positive correlation with the symptoms of near-related visual discomfort [5–7]. However, only few studies on accommodative changes after refractive surgery have been published [8–10]. In addition, they mainly focused on accommodative amplitude and accommodative facility changes. Except for accommodative amplitude and accommodative facility, an alternative way to assess accommodation is to measure the stimulus response function [11]. By measuring the accommodative responses to different stimulus levels, we can get the information about accommodative lag that is associated with symptoms of near-related visual discomfort [5–7].

To date, the changes of accommodation in SMILE procedure has never been reported before. In this study, we studied the accommodative response and accommodative lag changes after SMILE for moderate to high myopia correction.

## Methods

### Subjects

In this prospective, non-randomized study, 32 patients (9 male and 23 female) who underwent SMILE between January and February 2015 at Refractive Surgery Center of the Department of Ophthalmology, Eye and ENT Hospital of Fudan University were enrolled. Inclusion criteria included ages 18–30, corrected distance visual acuity (CDVA) of 20/20 or better, stable refraction for 2 years. Old subjects may have difficulty in maintaining stable accommodation at large stimulus levels which maybe usually closer than their near point [12]. Therefore, patients older than 30 were excluded. Patients with strabismus were excluded as well and the near phoria (at 33 cm) was measured by modified thorington phoria test. Patients with systemic diseases known to affect accommodation such as multiple sclerosis, Graves disease, myasthenia gravis or Parkinson disease were also excluded.

This study followed the tenets of the Declaration of Helsinki and was approved by the ethics committee of the Eye and ENT Hospital of Fudan University. Informed written consent was obtained from all participants.

### Surgical procedure

The VisuMax femtosecond laser system (Carl Zeiss Meditec, Jena, Germany) with a repetition rate of 500 kHz and pulse energy of 130 nJ was used to perform SMILE. Standard SMILE procedures were performed by the same surgeon (XTZ) for all the patients. Medication was received as follows: ophthalmic solution of levofloxacin, 0.1 % fluorometholone solution, and non-preservative artificial tear (carboxymethylcellulose sodium eye drops; Allergan, Inc., Irvine, CA).

### Accommodative changes measurement

Open-field autofractor was used to measure accommodative response and it is more objective compared with monocular estimate method (MEM) and Nott retinoscopy [13]. The accommodative response was obtained viewing monocularly with spherical equivalent refractive error corrected, using an open-field autorefractor (Grand Seiko WAM-5500, Japan) at different stimulus levels (2.00 diopters (D), 2.50D, 3.00D, 4.00D and 5.00D) for the right eyes before a standard SMILE surgery and at 1-month follow-up after surgery. Since there is no significant difference in accommodative lag between eyes [14, 15], the right eye was chosen as the previous studies [11, 13]. Subjects were instructed to keep looking attentively (blur-free) at a high contrast optotype (20/100 letters) at different viewing distances (50 cm, 40 cm, 33 cm, 25 cm, 20 cm, sequentially). The left eye was occluded and measurements were made with the room lights off. The patient’s right eye viewed the target through the trial frame. The patient was instructed to look at the central letter and to keep it clear. Using the joystick to maintain focus of the corneal reflections on the monitor, the same examiner (KZ) took all the accommodation measurements. The sign of the spherical equivalent accommodation reading was changed (plus to minus or minus to plus) to yield the accommodative response, which when subtracted from the accommodative demand yielded the accommodative lag if positive or the accommodative lead if negative. Five measurements were then taken by autorefraction.

### Data analysis

The accommodative lag/lead measurements used for analysis were the median value calculated from the five spherical equivalent measures of accommodative response by autorefraction. All statistical analysis was performed using the Statistical Package for Social Sciences (SPSS, Version 20) and reported as mean ± standard deviation. The paired t test was used for comparisons between the preoperative and postoperative data. Multiple linear regression model was conducted to evaluate factors associated postoperative accommodative lag after SMILE with postoperative accommodative lag. For all tests, a *P* < 0.05 was defined as statistically significant.

## Results

The mean age of the patients were 23.34 ± 2.90 years and the mean preoperative manifest refraction spherical equivalent was −5.74 ± 1.98 diopters. At near (33 cm), esophoria > 1 prism diopter (PD) was present in 6.24 % of patients, exophoria > 1 PD in 59.38 %, and orthophoria in 34.38 %. The patient information and baseline data are shown in Table 1. The preoperative and postoperative uncorrected near visual acuity (UNVA) at 33 cm of all patients was 20/20 or better. No patient has complained of near-related visual discomfort in one-month post-operation.

**Table 1:** Demographic data

Variables	Mean	SD	Range
Age (y)	23.34	2.90	18 to 29
Preoperative MRSE (D)	−5.74	1.98	−10.50 to −3.00
MRSE changes (D)	−5.77	1.85	−10.25 to −2.88
Postoperative MRSE (D)	−0.03	0.30	−0.63 to +0.63

*﻿y* years﻿, *MRSE* manifest refractive spherical equivalent, *D* diopters

**Fig. 1:**
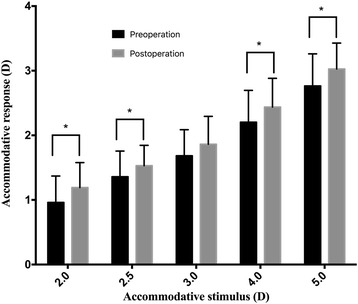
Preoperative and postoperative accommodative responses to different stimulus levels. A significant difference was detected between the preoperative and postoperative data at different stimulus levels (2.00D: *P* = 0.022; 2.50D: *P* = 0.046; 3.00D: *P* = 0.060; 4.00D: *P* = 0.030 and 5.00D: *P* = 0.009)

**Fig. 2:**
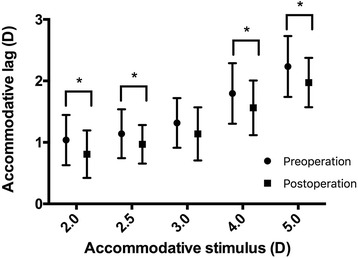
Preoperative and postoperative accommodative lags to different stimulus levels. The accommodative lag for each target stimulus except for 3.00D was significantly smaller in the postoperative group compared with preoperative group. (2.00D; *P* = 0.022, 2.50D; *P* = 0.046, 3.00D; *P* = 0.060, 4.00D; *P* = 0.030 and 5.00D; *P* = 0.009)

Figures 1 and 2 show the preoperative and postoperative accommodative responses and lags to different stimulus levels (2.00D, 2.50D, 3.00D, 4.00D and 5.00D). The accommodative lag for each target stimulus except for 3.00D was significantly smaller in the postoperative group compared with preoperative group. The average of accommodative lags in postoperative group (1.29 ± 0.58) was significantly lower than in the preoperative group (1.51 ± 0.63) (*P* < 0.001). Significant differences were detected both preoperative and postoperative accommodative responses and lags to different stimulus levels (responses: preoperative group: F = 88.90, *P* < 0.001, postoperative group: F = 107.82, *P* < 0.001; lags: preoperative group: F = 41.10, *P* < 0.001, postoperative group: F = 45.38, *P* < 0.001). Both accommodative response and lag became significantly larger at lager stimulus.

Multiple linear regression model analysis in Table 2 reveals that postoperative accommodative lag after SMILE increased as preoperative manifest refractive spherical equivalent increased (*P* = 0.006) and preoperative accommodative lag increased (*P* = 0.04).

**Table 2:** Factors associated with postoperative accommodative lag after smile in multiple regression analysis

Variables	b	*P*
Preoperative MRSE (D)	0.41	0.006
Preoperative AL (D)	0.29	0.04

*b* regression coefficient, *MSRE* manifest refractive spherical equivalent, *AL* accommodative lag, *D* diopters

## Discussion

Under viewing conditions at near distances with a steady near point stimulus, a small accommodative lag will be present [16]. Accommodative lag is quite common. Most individuals fail to bring the target into complete focus on the retina due to accommodative lag [17]. It is believed that some discrepancy between accommodative demand and accommodative response is normal though no criteria has been made yet.

In this study, we found that at closer viewing distances, accommodative lags and response became larger. Accommodative response and lag typically increased with increasing demand. It matched the accommodative classical model which was also reported by others [13, 18, 19].

Our results indicated that the accommodative changes after SMILE was apparent (*P* < 0.001). The accommodative response increased and accommodative lag decreased after SMILE (*P* < 0.001). Previous studies concerning other parameters to measure accommodation also reported that accommodation after refractive surgery was improved obviously compared with preoperative accommodation. Karimian et al. determined that accommodative facility increased in myopic patients 1 month after photorefractive keratectomy (PRK) [8]. Moreover, Liu et al. and Fu et al. observed an increase of accommodative amplitude and accommodative facility 1 month after implantation of iris-fixated phakic intraocular lens respectively [9, 10]. The lower accommodative lag that was found after SMILE could be an early sign of an addition in the accommodation amplitude [20]. Thus, our results are in accordance with previous studies.

Multiple linear regression model analysis revealed that preoperative manifest refractive spherical equivalent (*P* = 0.006) and preoperative accommodative lag (*P* = 0.04) showed a significant impact on postoperative accommodative lag. The findings suggested that patients with higher preoperative manifest refractive spherical equivalent, higher preoperative accommodative lag tended to have higher postoperative lag. Karimian et al. also reported in PRK that preoperative accommodative amplitude and accommodative facility had a significant effect on 3-months postoperative accommodative amplitude and accommodative facility, respectively [8].

Previous studies showed that accommodative lag played an important role in visual discomfort symptom [5–7]. Visual discomfort describes adverse symptoms associated with reading and close work, containing headaches, asthenopia, diplopia, light sensitivity, blurred text, moving letters and other unpleasant somatic symptoms and perceptual distortions [21]. The incidence of visual discomfort symptom is very high. Borsting et al. found that 60 % of surveyed college students with moderate to severe symptoms [22]. Many studies confirmed that an increase in accommodative lag was found in the high visual discomfort patients, whereas the low discomfort group had a stable response [5–7]. Accommodative lag accounts for a proportion of symptom variance, while accommodative amplitude is a poor predictor of visual discomfort [5, 23]. Chase et al. concluded that the increase of accommodative lag developing at a viewing distance of 20 cm was better at predicting visual discomfort symptoms than clinical testing [5]. In this study, postoperative UNVA at 33 cm of all patients was 20/20 or better and no patient has complained of near-related visual discomfort in one-month post-operation. We hypothesize that the decrease in accommodative lag after SMILE procedure might relieve the visual discomfort symptom, which might contribute to patients’ satisfaction of SMILE.

There are some limitations in this study. The follow-up period of the study was not long enough to judge about the course of accommodative response and accommodative lag changes after surgery. In addition, we did not objectively evaluate frequency and severity of visual discomfort symptom using a survey in the patients. Near-related visual discomfort might be negligible to some patients. It would be better if visual discomfort was evaluated with a survey developed by Convergence Insufficiency Treatment Trial Group and the other developed by Conlon et al. [24].

## Conclusions

In conclusion, this is the first report of accommodative response and accommodative lag changes after SMILE. Our preliminary results suggested that a decrease in postoperative accommodative lag might be related to the relief of the visual discomfort symptom. It might help refractive surgeon to provide the possible guidance for postoperative consult in clinical practice.

## Abbreviations

CCL: Continuous curvilinear lenticulerrhexis
CDVA: Corrected distance visual acuity
D: Diopters
MEM: Monocular estimate method
PD: Prism diopter
PRK: Photorefractive keratectomy
SMILE: Small incision lenticule extraction
SPSS: Statistical package for social sciences

## References

[1] Shah R, Shah S, Sengupta S (2011). Results of small incision lenticule extraction: All-in-one femtosecond laser refractive surgery. J Cataract Refract Surg.

[2] Sekundo W, Kunert KS, Blum M (2011). Small incision corneal refractive surgery using the small incision lenticule extraction (SMILE) procedure for the correction of myopia and myopic astigmatism: results of a 6 month prospective study. Br J Ophthalmol.

[3] Zhao Y, Li M, Yao P, Shah R, Knorz MC, Zhou X (2015). Development of the continuous curvilinear lenticulerrhexis technique for small incision lenticule extraction. J Refract Surg.

[4] Ivarsen A, Asp S, Hjortdal J (2014). Safety and complications of more than 1500 small-incision lenticule extraction procedures. Ophthalmology.

[5] Chase C, Tosha C, Borsting E, Ridder WH (2009). Visual discomfort and objective measures of static accommodation. Optom Vis Sci.

[6] Momeni-Moghaddam H, Goss DA, Sobhani M (2014). Accommodative response under monocular and binocular conditions as a function of phoria in symptomatic and asymptomatic subjects. Clin Exp Optom.

[7] Tosha C, Borsting E, Ridder WH, Chase C (2009). Accommodation response and visual discomfort. Ophthalmic Physiol Opt.

[8] Karimian F, Baradaran-Rafii A, Bagheri A, Eslani M, Bayat H, Aramesh S, Yaseri M, Amin-Shokravi A (2010). Accommodative changes after photorefractive keratectomy in myopic eyes. Optom Vis Sci.

[9] Liu LN, Lu F, Wang QM, Xue AQ, Chen SH, Chen HB (2010). Change of accommodative function in phakic eyes with iris-fixated phakic intraocular lens implantation. Zhonghua Yan Ke Za Zhi.

[10] Fu J, Wang XZ, Wang NL, Wang JH, Zhao SQ (2013). Accommodation perimeters after phakic posterior chamber implantable contact lens implantation. Zhonghua Yan Ke Za Zhi.

[11] Wick B, Hall P (1987). Relation among accommodative facility, lag, and amplitude in elementary school children. Am J Optom Physiol Opt.

[12] Kalsi M, Heron G, Charman WN (2001). Changes in the static accommodation response with age. Ophthalmic Physiol Opt.

[13] Manny RE, Chandler DL, Scheiman MM, Gwiazda JE, Cotter SA, Everett DF, Holmes JM, Hyman LG, Kulp MT, Lyon DW, Marsh-Tootle W, Matta N, Melia BM, Norton TT, Repka MX, Silbert DI, Weissberg EM, Correction of Myopia Evaluation Trial 2 Study Group for the Pediatric Eye Disease Investigator G (2009). Accommodative lag by autorefraction and two dynamic retinoscopy methods. Optom Vis Sci.

[14] Momeni-Moghaddam H, McAlinden C, Azimi A, Sobhani M, Skiadaresi E (2014). Comparing accommodative function between the dominant and non-dominant eye. Graefes Arch Clin Exp Ophthalmol.

[15] del Pilar CM, Garcia-Munoz A, Garcia-Bernabeu JR, Lopez A (1999). Comparison between MEM and Nott dynamic retinoscopy. Optom Vis Sci.

[16] Garcia A, Cacho P (2002). MEM and Nott dynamic retinoscopy in patients with disorders of vergence and accommodation. Ophthalmic Physiol Opt.

[17] Gwiazda J, Thorn F, Bauer J, Held R (1993). Myopic children show insufficient accommodative response to blur. Invest Ophthalmol Vis Sci.

[18] Nakatsuka C, Hasebe S, Nonaka F, Ohtsuki H (2005). Accommodative lag under habitual seeing conditions: comparison between myopic and emmetropic children. Jpn J Ophthalmol.

[19] Rosenfield M, Gilmartin B (1990). Effect of target proximity on the open-loop accommodative response. Optom Vis Sci.

[20] Jimenez R, Martinez-Almeida L, Salas C, Ortiz C (2011). Contact lenses vs spectacles in myopes: is there any difference in accommodative and binocular function?. Graefes Arch Clin Exp Ophthalmol.

[21] Borsting E, Chase C, Tosha C, Ridder WH (2008). Longitudinal study of visual discomfort symptoms in college students. Optom Vis Sci.

[22] Borsting E, Chase CH, Ridder WH (2007). Measuring visual discomfort in college students. Optom Vis Sci.

[23] Borsting E, Tosha C, Chase C, Ridder WH (2010). Measuring near-induced transient myopia in college students with visual discomfort. Optom Vis Sci.

[24] Drew SA, Borsting E, Escobar AE, Liu C, Castellanos E, Chase C (2013). Can chronic visual discomfort measures accurately predict acute symptoms?. Optom Vis Sci.

